# Maximizing the utility of public data

**DOI:** 10.3389/fgene.2023.1106631

**Published:** 2023-03-31

**Authors:** Mahmoud Ahmed, Hyun Joon Kim, Deok Ryong Kim

**Affiliations:** ^1^ Department of Biochemistry and Convergence Medical Sciences, Institute of Health Sciences, College of Medicine, Gyeongsang National University, Jinju, Republic of Korea; ^2^ Department of Anatomy and Convergence Medical Sciences, Institute of Health Sciences, College of Medicine, Gyeongsang National University, Jinju, Republic of Korea

**Keywords:** public-data, data-reuse, data-analysis, data-sharing, reproducible-research

## Abstract

The human genome project galvanized the scientific community around an ambitious goal. Upon completion, the project delivered several discoveries, and a new era of research commenced. More importantly, novel technologies and analysis methods materialized during the project period. The cost reduction allowed many more labs to generate high-throughput datasets. The project also served as a model for other extensive collaborations that generated large datasets. These datasets were made public and continue to accumulate in repositories. As a result, the scientific community should consider how these data can be utilized effectively for the purposes of research and the public good. A dataset can be re-analyzed, curated, or integrated with other forms of data to enhance its utility. We highlight three important areas to achieve this goal in this brief perspective. We also emphasize the critical requirements for these strategies to be successful. We draw on our own experience and others in using publicly available datasets to support, develop, and extend our research interest. Finally, we underline the beneficiaries and discuss some risks involved in data reuse.

## Introduction

The human genome project (HGP) galvanized the scientific community around an ambitious goal (Lander et al., 2001). Upon completion, the HGP produced several crucial discoveries, and a new era of research began (Gates et al., 2021). The project provided an estimate of the number of genes and a comprehensive list of their coding sequences. These developments have allowed for a shift away from single gene models and kickstarted the discipline of systems biology analysis. Furthermore, the non-coding regions came into focus, and their function began to be studied along with the variation across individuals (ENCODE Project Consortium TEP Feingold et al., 2004; Sabeti et al., 2007). More significantly, scientists developed new technologies and analytic methods during the period of the project. The cost reduction allowed many more labs to generate high-throughput datasets (Metzker, 2010). The project also served as a model for other big collaborations that generated larger datasets. These included efforts to sequence a large number of genomes from different populations across the globe (Auton et al., 2015; Wall et al., 2019; Smedley et al., 2022). Others concentrated on specific diseases, and disease models such as cancer (Weinstein et al., 2013; Subramanian et al., 2017).

Alongside these ambitious endeavors, small datasets were generated and made public and continued to accumulate in repositories. The gene expression omnibus (GEO), ArrayExpress, and sequence read archive (SRA) are just a few examples (Edgar et al., 2002; Parkinson et al., 2007; Leinonen et al., 2011). Individual labs typically use these repositories to document and distribute raw and processed data accompanying publications. As a result, the scientific community should consider how to use these resources for research and the public good. Efforts went into fostering the adoption of best practices to document and share data, and proper policies around accessibility (Thorogood and Knoppers, 2016; Rehm et al., 2021). Indeed, recognizing reuse as a legitimate form of research at the junior and senior levels has become more acceptable and encouraged (Duvallet, 2020; Raman, 2021). Furthermore, initiatives are being proposed to develop cloud environments to store, manage, and analyze data in effective, scalable, and secure ways (Schatz et al., 2022). Similarly, models have been developed to create an ecosystem to improve data description and hosting (Charbonneau et al., 2022).

**FIGURE 1:**
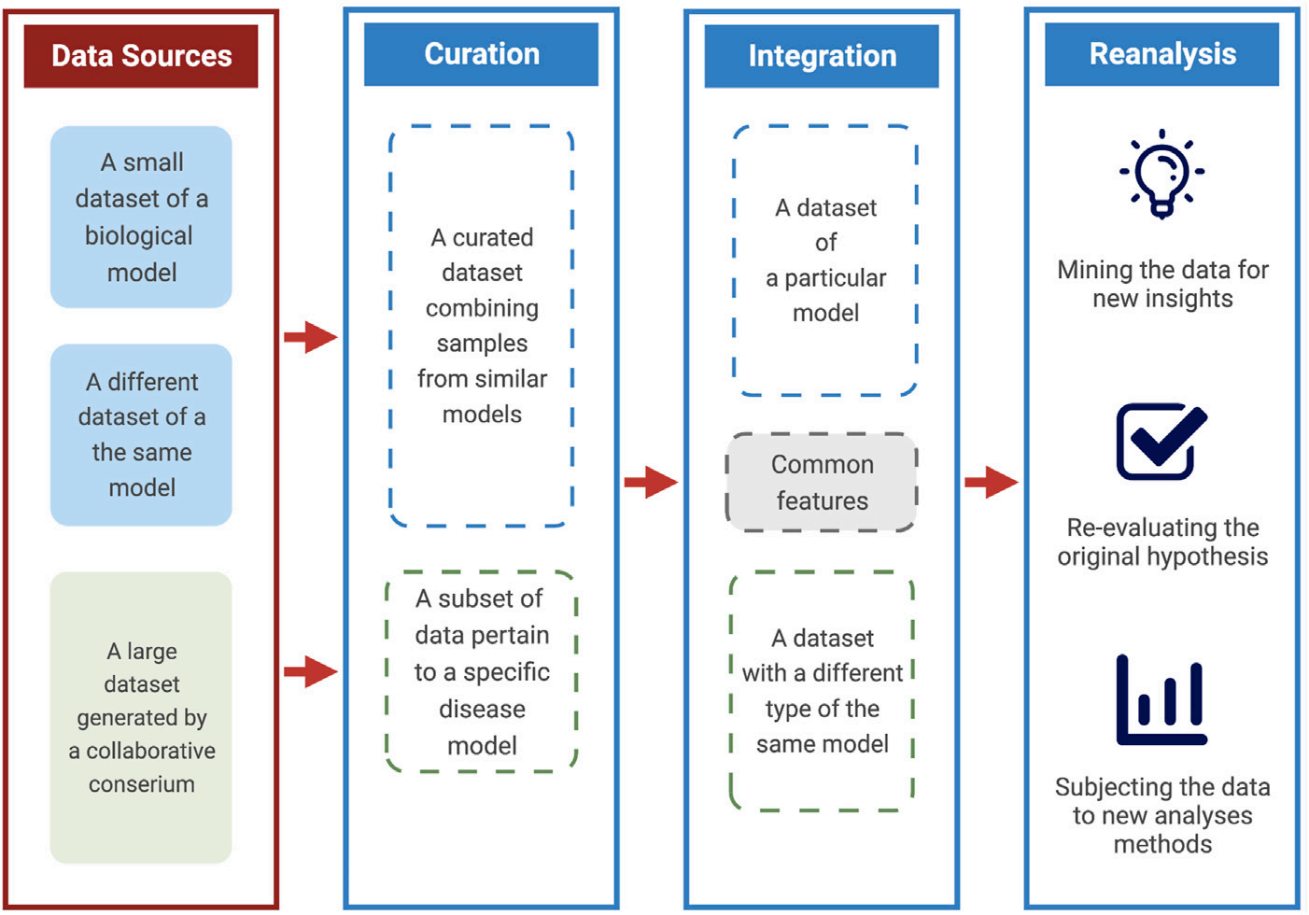
A scheme for maximizing the utility of public data. A diagram explaining the ways and benefits of using data from public sources. These include curating datasets of particular models, integrating different data types, and re-analysis with various goals.

In this short perspective, we highlight three critical areas to maximize the utility of publicly available data. We draw on ower own experience and others in using genomics and transcriptomic public datasets to support and extend our research interest. A dataset can be re-analyzed, curated, or integrated with other forms of data to enhance its utility (Figure 1). Although not an exhaustive list of possible use cases, those three strategies encapsulate a majority of research types that rely on the existence of public data. Furthermore, each has identifiable benefits and requirements. Finally, we remark on the overall benefit of reuse, the primary beneficiaries, and highlight some of its risks.

### Reanalyzing primary data

High-throughput experiments generate simultaneous measurements of a large portion, if not all, of the genome. It has become a standard practice for researchers to share the raw data and documentation of how they generated it. The obvious case for reuse is to mine the dataset for insights that were not explored in the initially published studies. Investigators could focus on a particular subset of the data and analyze it in-depth. When data is available in raw or unaltered formats. The availability of the data facilitates the peer-review process where the suggested hypothesis can be examined and the analysis reproduced. Others choose to verify or refute the hypothesis presented in the original analyses by examining them independently. Both reuse cases yield additional value and benefit the wider community. We employed existing gene expression, and DNA binding datasets of preadipocytes to explore the role of autophagy during adipocyte differentiation (Ahmed et al., 2019). Our analysis focused on the subset of gene products involved in lipogenesis and autophagy. We were able to link the regulation of key autophagy genes to the transcription factors that drive the reprogramming of progenitor cells into mature adipocytes.

Any given dataset can only be analyzed in a few ways in any given study. Existing and newly developed tools can be applied to these datasets to generate new insights. Often, statistically sophisticated approaches have the potential to extract more information from the same data points. We used co-expression analysis and unsupervised learning methods to study gene product interactions in the preadipocyte differentiation dataset mentioned above (Ahmed et al., 2018). Furthermore, we deconvoluted the mixture of differentiating adipocytes into subpopulations. We suggested that mature adipocytes originate from a small fraction of the progenitors (Ahmed et al., 2021a). Researchers have employed large datasets of RNA-seq to predict genes, transcripts, and promoters (Steijger et al., 2013; Keilwagen et al., 2018; Wilson et al., 2021). Analysis methods such as chromatin segmentation are only possible when a considerable number of histone markers have been profiled using ChIP-seq (Hoffman et al., 2012). These and others are examples of research pursuits that are only possible because or enhance the utility of publicly available data.

### Curating data from different sources

Researchers interested in a particular topic often use similar models and similar experimental designs. Despite being generated using different protocols, combining the datasets from separate groups could help filling the gaps in the design and *increasing the statistical power* of the analysis. For an example from our work, the cell line model 3T3-L1 has been used for years in standard time-course experiments to study adipocyte differentiation. Combining several gene expression experiments produced a dataset with numerous samples and covered more time points in the differentiation course (Ahmed and Kim, 2019). A similar problem arises when generating gene expression data with drug or genetic perturbations. Incorporating more than one dataset means including additional perturbations in the study (Ahmed et al., 2020a). The reverse is also possible by curating and annotating a subset of a larger dataset to address a specific aspect of the model or focus on a data type (Ganzfried et al., 2013).

Curation in either direction, smaller to larger, or larger to smaller datasets improves the utility of the data. One added benefit is that curators have to homogenize data from different sources and use unified terminologies. Furthermore, curators can pre-process and quality assesses large files of row data and make the data available in more accessible formats. The recount3 project accomplished just that by curating and processing thousands of RNA-seq reads files and making the results available in the form of gene counts (Wilks et al., 2021). Likewise, KnockTF is a curated gene expression dataset of transcription factors knockdown experiments (Feng et al., 2019). These two examples highlight yet another advantage of curation. Namely, it exposes the data to the scientific community beyond computational labs and makes it available and easy to use for lab biologists.

### Integrating multiple types of data

Different high-throughput technologies generate data types that describe different layers of biology. Integrating data types can be beneficial to either verify or complement the observations made based on a single data type. For example, the binding of a transcription factor to the DNA of a specific region is not necessarily a claim about the function of that transcription factor. However, the likelihood that this binding is functional increases if, under the perturbation of that transcription factor, the expression of the nearest gene changes. We used both binding and gene expression data to study the interaction between adipogenic transcription factors and the autophagy genes of interest. We were able to show that a hierarchy of transcription regulators, including those controlling the differentiation program, regulates autophagy directly or indirectly through other factors (Ahmed et al., 2022). Integrating data types, in this case, allowed for identifying a phenomenon that was not otherwise obvious from gene expression or DNA-binding alone.

New methods capitalize on this idea of combining data from different sources. For example, binding and expression target analysis (BETA) infers direct target genes of transcription factors by integrating binding peaks, and gene expression changes under the factor’s perturbation (Wang et al., 2013). We further extended this method to the interaction of two DNA-binding proteins as they function in cooperative or competitive ways to induce or repress a shared target (Ahmed et al., 2020b). Existing biological knowledge can also help in modeling and interpreting experimental data. The known pathways can be encoded in a network where the nodes are the biological entities, and the edges are the known interactions between them. The biological expression language (BEL) is one way to represent this knowledge in a standard computable graph (Hoyt et al., 2018). Methods such as network perturbation amplitudes (NPA) take advantage of these graphs to infer the function of the biological entities from the changes in gene expression in response to drug treatment or genetic perturbations (Martin et al., 2012; Martin et al., 2019). We used this approach to generate a database of cancer cell-specific perturbations and to screen for potential antimetastatic drugs in breast cancer (Ahmed and Kim, 2021). In one instance, we inferred the pathways that control the expression of an antimetastatic gene along with multiple drugs that target it (Ahmed et al., 2021b). We experimentally validated some of these predictions.

## Discussion

In this perspective, we highlighted three avenues to maximize the utility of existing public data. Namely, re-analyzing primary data, curating data from different sources, and integrating multiple data types. We also opined the potential benefits of each strategy with examples from our work and others. We acknowledge that successful reuse places demands on the broader community regarding the documenting and sharing of data (Table 1). Other broad benefits can be accrued from this model of sharing and reuse, which we discuss next in addition to the risks some pointed out.

**TABLE 1:** Ways, benefits, and requirements to increase the utility of public data.

Strategy	Benefits	Requirements
Reanalysis	Mining the data for new insights	• Sharing the raw data
		• Documenting and sharing meta-data
	Re-evaluating the original hypothesis	• Clear presentation of how data were generated and analyzed
		• Sharing reproducible code
	Subjecting the data to new analyses methods	• Encouraging the development of new analysis methods and techniques
		• Developing new tools
Curation	increasing the sample size	Proper documentation and sharing of experimental protocols
	Filling in the gaps	• Developing an ontology to code experimental variables
		• Transparent and transferable annotations
	Improving the utility	• Funding for computational resources to generate and maintain processed data
		• Lowering the entry barrier for lab biologists
Integration	Complementing an analysis	Encouraging collaboration between dry and wet labs
	Examining a phenomenon from different angles	Generating complementary datasets (OMICS)
	Spurring the development of new methods	Funding open-source methods and tools development

Our model of sharing and reusing data focuses on extracting more value from the available resources. However, we acknowledge that for this to be standard, it requires data to be documented and shared in transparent reproducible ways. In addition, resources should be available for curating, annotating, developing tools, and re-analyzing data. We similarly view these as stimulators for good: encouraging best practices of reproducible research and developing models for open science (Peng, 2011; OECD. Making Open Science a Reality, 2015). In other words, if data were generated with potential reuse, by the primary authors or others, in mind, it would be a net benefit to the community. The conditions that encourage reproducible open science are the same that foster and encourage reuse. We also believe data sharing and reuse would benefit researchers in low-resource labs and developing nations (Cheah et al., 2015). Finally, easily accessible data would facilitate and lower the entry barrier for non-computational researchers to use the extensive knowledge made possible by large datasets.

It is necessary to acknowledge the potential risks associated with the reuse of public data. Sielemann and colleagues underlined some of these and suggested solutions (Sielemann et al., 2020). They warn that user-submitted data may be of questionable quality and require substantial work to locate and obtain before reaching the point of analysis. This effort on behave of the researcher interested in reuse may be wasted as the data quality would only be assessed later in the process. Furthermore, reusing public data may produce duplicate records of the same dataset. Finally, several ethical issues arise in reuse cases. For example, not crediting the original authors may disincentive others from sharing their data and code in the future (Curty et al., 2017). Besides, funding for generating new datasets may stall because similar datasets exist or others could be repurposed (Nature, 2016).

## Data Availability

The original contributions presented in the study are included in the article/supplementary material, further inquiries can be directed to the corresponding author.
